# The survey of the status of self-stigma of depression and its relationship with demographic factors in Gonabad, Iran

**DOI:** 10.3389/fpsyt.2024.1463879

**Published:** 2024-11-28

**Authors:** Hadi Tehrani, Fatemehzahra Naddafi, Mahbobeh Nejatian, Alireza Jafari

**Affiliations:** ^1^ Social Determinants of Health Research Center, Mashhad University of Medical Sciences, Mashhad, Iran; ^2^ Department of Health Education and Health Promotion, School of Health, Mashhad University of Medical Sciences, Mashhad, Iran; ^3^ Student Research Committee, Gonabad University of Medical Sciences, Gonabad, Iran; ^4^ Social Determinants of Health Research Center, Gonabad University of Medical Sciences, Gonabad, Iran; ^5^ Department of Health Education and Health Promotion, School of Health, Social Development and Health Promotion Research Center, Gonabad University of Medical Sciences, Gonabad, Iran

**Keywords:** self-stigma, depression, stigma, mental health, help seeking

## Abstract

**Introduction:**

Depression is a common mental illness. Depression stigma can reduce individuals’ desire to seek mental health services. No study has investigated depression self-stigma and its relationship with demographic factors in the general population of Gonabad city in Iran. Therefore, this study was aimed at determining the relationship between depression self-stigma and demographic factors.

**Methods:**

This cross-sectional study was conducted among 1,075 Iranians living in Gonabad city in 2024. Proportionate stratified sampling was used to select participants. Data collection tools included demographic data and the self-stigma of depression scale (SSDS). Higher SSDS scores indicate greater depression self-stigma. Data were analyzed using SPSS 24 with the independent samples t-test, one-way analysis of variance, and Pearson correlation.

**Results:**

The mean (± SD) of self-stigma of depression (SSD) was 42.51 (9.31). Only 16.1% of the participants mentioned that they had a mental disorder, and 17.2% reported that they had been referred to a psychologist. Based on the results, males had more SSD (*p* = 0.028), help-seeking inhibition (*p* < 0.001), and shame (*p* = 0.002) than females. There were no significant relationships between education level, economic status, age group, occupation, and marital status with SSD (*p* > 0.05). Individuals with a history of mental disorder had higher SSD (*p* = 0.011) and help-seeking inhibition (*p* = 0.004). People who did not obtain information related to mental illness had more help-seeking inhibition (*p* = 0.001). Based on the Pearson correlation results, a positive and significant correlation coefficient was observed between the subscales of SSDS (*P* < 0.001).

**Conclusion:**

The results of the study showed that SSD level was 42.51 ± 9.31 from 70. SSD is one of the main obstacles to help-seeking and treatment, so providing knowledge and awareness in this area is essential to the community.

## Introduction

Depression is a major health problem and the most common mental disorder in the general population. This disorder is now an epidemic (1–3). About 322 million people worldwide struggle with depressive disorders (4) and approximately 5% of adults and 5.7% of the elderly (over 60 years old) in the world suffer from depression (5). The prevalence of depression in Iran is also increasing, and the results of a systematic review in Iran indicated a prevalence of depression was 34.26% (6).

Depression is characterized by loss of pleasure or interest, sadness, feelings of worthlessness or guilt, disturbance in appetite or sleep, poor concentration, and tiredness (2). Depression, as one of the main factors in the burden of disease and disability, causes significant decreases in productivity and quality of life, and in its most severe form, depression can lead to increased risks of mortality and suicide (3, 7–10).

Although depression can be debilitating and even fatal like diabetes or heart disease, it is highly stigmatized (11). The stigma of mental illness is a barrier to the implementation of necessary interventions to reduce the burden of depression (7). As a result, depressed patients suffer not only from their disabilities and symptoms but also from the stigma associated with depression. Stigma is a sign of scandal, shame, or disapproval. As a social process, stigma is characterized by rejection, separation, discrimination, discreditation, and blame toward a group or individual (12, 13). Self-blame is referred to as “the consequences one experiences are a direct result of one’s actions or character. In the context of behavioral medicine, this may be either beneficial or harmful depending on if it leads to positive behavior change or increased negative affectivity and lack of behavior change.” (14)

There are different types of stigmas. One type of stigma is self-stigma, which is created by internalizing negative beliefs and perceptions of a person suffering from mental illness and attributing them to themselves (15, 16). The results of a systematic review and meta-analysis indicated a 29% prevalence of depression self-stigma in the world (17). Self-stigma may jeopardize the process of seeking help and treatment and often causes a deep feeling of guilt and shame (16). Rickwood et al., defined help-seeking as “a term that is generally used to refer to the behavior of actively seeking help from other people. It is about communicating with other people to obtain help in terms of understanding, advice, information, treatment, and general support in response to a problem or distressing experience.” (18)

High self-stigma is associated with reduced social functioning, high psychological distress, and impaired quality of life (13). The results of a study on a population of patients with depression in the Czech Republic indicated a positive relationship between the level of self-stigma and the severity of disease symptoms and a negative relationship between the level of self-stigma and quality of life (19). In a study conducted in Iran, it was found that 26.7% of the participants experienced high to moderate levels of self-stigma in the population with bipolar disorder type I (presence of periods of major depression and mania) (20). In a study among Iranian students about depression stigma, women had a more positive attitude toward those suffering, but men believed that those suffering from depression are dangerous and should be isolated (21). Additionally, Iran is a developing country, and in developing countries, compared with developed countries, more stigma has been reported regarding mental illness (22, 23). For instance, in a study, the stigma of mental illness in Iran was reported 40% (24). In addition, in Iran, the use of mental health services is widely stigmatized in the general population because of the stigma associated with mental illnesses (22).

Overall, we conclude that the prevalence of depression in Iran is increasing and depression self-stigma is a potential obstacle to seeking help. However, to the best of our knowledge, no study has investigated depression self-stigma and its relationship with demographic factors in the general population of Iran. If our goal is to implement early interventions and treat depression, the concept of stigma of depression should be given more attention (25). Finding factors related to stigma can also be a key approach toward developing general interventions and strategies to reduce stigma in the population of people with depression (26). Therefore, this study was conducted with the aim of surveying the level of self-stigma of depression (SSD) and investigating its relationship with demographic factors in Gonabad city, Iran.

## Method

This cross-sectional study was performed to survey the status of SSD and its relationship with demographic factors in the Iranian population in Gonabad city (located in the northeast of Iran), 2024.

### Sample size

According to the results of the study (27), the standard deviation of stigma of depression was 4.8, with a 95% confidence level, 80% power test, 0.48 accuracy, and nonresponse rates of 25%, sample size was calculated at least 1,045 participants and finally we selected 1075 participants.


$$n=\frac{{\left(z_{1-\frac{\alpha }{2}}+z_{1-\beta }\right)}^{2}\;{\left(S\right)}^{2}\;}{{\left(d\right)}^{2}}=\frac{{\left(1.96+0.84\right)}^{2}\;{\left(4.8\right)}^{2}\;}{{\left(0.48\right)}^{2}}$$

### Sampling method

The sample size required for this study was selected using the proportional stratified sampling method, and participants were recruited from comprehensive community health centers in Gonabad city (*n* = 3). In Iran, electronic health records are usually kept at a comprehensive community health center. Then, each center was considered a stratum, and the population aged 18 years and older was determined in each center. In the next step, participants were selected by simple random sampling and based on the inclusion criteria from each center. Intent to participate in the study, to be a resident of Gonabad city, aged 18 years and older, and lack of specific cognitive problem (based on the electronic health records) to complete the questionnaire were the inclusion criteria in this study. Unwilling to continue completing the questionnaire or questionnaires with more than 20% missing data were the exclusion criteria in this study. To complete the questionnaires, the questioner referred to comprehensive community health centers and delivered questionnaires to participants, and questionnaires were completed by self-report. Only questionnaires for illiterate people were completed using the interview method by questioner.

### Data collection tools

The questionnaire used in this study comprised two parts. The first part was related to demographic information, and the second part was related to the self-stigma of depression scale (SSDS).

- **Demographic part and condition of participants:** In this part, age group, economic status, occupation, education level, and so forth were assessed.
- **SSDS:** Barney et al., created this instrument (28). This scale is asses by 16 items and 4 subscales of social inadequacy with 4 questions, self-blame with 4 questions, help-seeking inhibition with 4 questions, and shame with 4 questions (28). The modified Persian version of SSDS was approved with 14 items and four subscales of social inadequacy (3 questions), self-blame (3 questions), help-seeking inhibition (4 questions), and shame (4 questions) among the Iranian population (29). In the Persian version of SSDS, two items were deleted with factor loading less than 0.4 in the confirmatory factor analysis section and the final model was approved with 14 items. The CVR and CVI of the Persian version of SSDS were 0.773 and 0.923, respectively. Cronbach α coefficient of SSDS and the subscales of social inadequacy, self-blame, help-seeking inhibition, and shame were 0.850, 0.874, 0.784, 0.813, and 0.861, respectively (29). SSDS is measured with 5 Likert scale (completely agree, agree, not agree and not disagree, disagree, completely disagree), and the score range of the Persian version of SSDS is 14–70, and higher scores indicate more self-stigma of depression (29).

### Statistical analysis

Data were entered and analyzed using SPSS 24 with a *p*-value of less than 0.05. The independent samples t-test was used to compare variables such as sex, education level, get information related to mental illness, referral to a psychologist, history of mental disorder with SSD, social inadequacy, help-seeking inhibition, self-blame, and shame. A one-way analysis of variance (one-way ANOVA) was used to compare age group, economic status, occupation, marital status, sources of obtaining health information, sources of obtaining information related to mental illness, and participant’s family referral to a psychologist with SSD, social inadequacy, help-seeking inhibition, self-blame, and shame.

## Results

**Figure 1:**
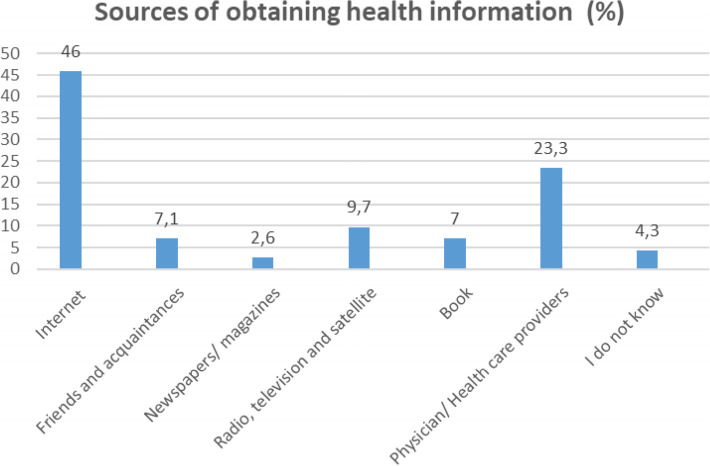
Results of sources of obtaining health information.

**Figure 2:**
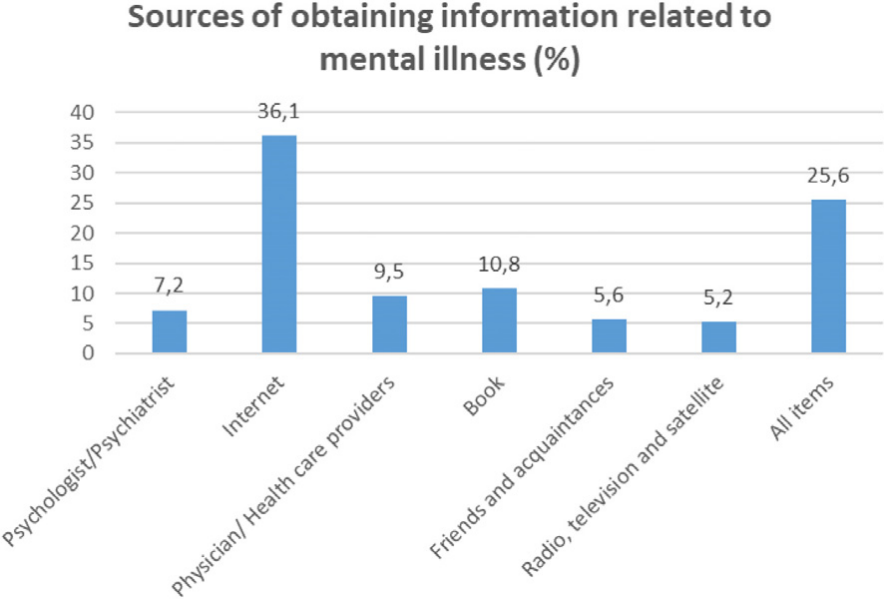
Results of sources of obtaining information related to mental illness.

Most of people were in the age group of 18–27 years (*n* = 432, 40.2%), and the mean age (± SD) of participants was 34.15 (13.05). 50.5% (*n* = 543) of people were female, 59.3% (*n* = 638) were married, and 62.7% (*n* = 674) had academic degree. Most participants (*n* = 495, 46%) reported that they got health information from the internet (**Table 1**, **Figure 1**). 74% (*n* = 796) of participants reported that they got information related to mental illness, and the internet (*n* = 297, 27.6%) was the most source to obtain this information (**Table 1**, **Figure 2**). Only 16.1% (*n* = 173) of participants mentioned that they had a mental disorder, and 17.2% (*n* = 185) reported that they referred to a psychologist so far. The more information was mentioned in **Table 1**.

**Table 1:** Characteristics of demographic variables.

Variables		*n* = 1,075	
		*n*	%
**Age group**	18–27	432	40.2
	28–37	243	22.6
	38–47	201	18.7
	> 47	199	18.5
**Sex**	Male	532	49.5
	Female	543	50.5
**Economic status**	Good	229	21.3
	Medium	711	66.1
	Weak	135	12.6
**Occupation**	Self-employed	162	15.1
	Employed	301	28
	Retired	60	5.6
	Housewife	122	11.3
	labor	29	2.7
	University students	380	35.3
	Unemployed	21	2
**Education level**	Diploma and low degree	401	37.3
	Academic degree	674	62.7
**Marital status**	Married	638	59.3
	Single	412	38.3
	Divorce	25	2.3
**Get information related to mental illness**	Yes	796	74
	No	279	26
**Sources of obtaining health information**	Internet	495	46
	Friends and acquaintances	76	7.1
	Newspapers/magazines	28	2.6
	Radio, television, and satellite	105	9.7
	Book	75	7
	Physician/Healthcare providers	250	23.3
	I do not know	46	4.3
**Sources of obtaining information related to mental illness**	Psychologist/Psychiatrist	59	7.2
	Internet	297	36.1
	Physician/Healthcare providers	78	9.5
	Book	89	10.8
	Friends and acquaintances	46	5.6
	Radio, television, and satellite	43	5.2
	All items	211	25.6
**Did you refer to psychologist?**	Yes	185	17.2
	No	890	82.8
**Have you ever had mental disorder?**	Yes	173	16.1
	No	901	83.9
**Did your family refer to psychologist?**	Yes	185	17.2
	No	710	66
	I don’t know	180	16.7

In **Table 2**, relationships between demographic variables and SSD were mentioned. Results of ANOVA showed that age group had a significant relationship with subscale of help-seeking inhibition, and the age group of more than 47 years had more help-seeking inhibition. Also, results showed that the age group 18–27 year had more self-blame. Results of an independent sample t-test showed that males had more SSD (*p* = 0.028), help-seeking inhibition (*p* < 0.001), and shame (*p* = 0.002) than females. Females only had more social inadequacy (*p* = 0.018) and self-blame (*p* = 0.016) than males. There was no significant relationship between the economic status with SSD and its subscales (*P* > 0.05). Results showed a significant relationship between marital status and help seeking inhibition. There was no significant relationship between the occupation with SSD and its subscales (*P* > 0.05). Also, there was no observed significant relationship between education level with SSD and its subscales (*P* > 0.05) (**Table 2**).

**Table 2:** Relationship between demographic variables and self-stigma of depression (SSD).

Variables		SSD *Mean (SD)*									
		Social inadequacy	*P*-value	Help-seeking inhibition	*P*-value	Self-Blame	*P*-value	Shame	*P*-value	SSD	*P*-value
Age group**	18–27	9.78 (2.72)	0.862	10.93 (3.50)	0.001	10.28 (2.39)	0.008	11.47 (3.68)	0.331	42.48 (8.69)	0.101
	28–37	9.60 (2.59)		11.28 (3.29)		9.60 (2.60)		11.27 (3.69)		41.77 (8.77)	
	38–47	9.70 (2.77)		11.12 (3.74)		10.21 (2.51)		11.05 (3.87)		42.09 (9.64)	
	> 47	9.75 (2.86)		12.21 (3.87)		10.19 (2.92)		11.71 (4.23)		43.88 (10.75)	
Sex*	Male	9.52 (2.74)	0.018	11.94 (3.63)	< 0.001	9.91 (2.64)	0.016	11.76 (3.93)	0.002	43.14 (9.76)	0.028
	Female	9.92 (2.69)		10.64 (3.45)		10.29 (2.50)		11.03 (3.69)		41.89 (8.81)	
Economic status**	Good	9.50 (2.89)	0.268	11.01 (3.72)	0.035	10.13 (2.64)	0.502	11 (3.82)	0.211	41.65 (9.66)	0.283
	Medium	9.82 (2.65)		11.23 (3.51)		10.13 (2.55)		11.50 (3.82)		42.70 (9.19)	
	Weak	9.60 (2.84)		12 (3.79)		9.85 (2.64)		11.49 (3.87)		42.95 (9.31)	
Occupation**	Self-employed	9.54 (2.57)	0.583	11.81 (3.48)	0.124	9.69 (2.70)	0.050	11.65 (3.79)	0.270	42.71 (8.77)	0.612
	Employed	9.73 (2.74)		11.37 (3.60)		10.14 (2.57)		11.27 (3.91)		42.53 (9.75)	
	Retired	9.76 (2.70)		11.93 (3.73)		10.21 (3.08)		11.90 (4.28)		43.81 (11.23)	
	Housewife	9.45 (2.84)		11.21 (3.85)		10.17 (2.70)		10.81 (3.91)		41.65 (9.68)	
	labor	9.24 (2.50)		11.37 (3.83)		10.13 (2.51)		11.03 (3.91)		41.79 (8.93)	
	University students	9.88 (2.76)		10.94 (3.52)		10.27 (2.36)		11.57 (3.67)		42.68 (8.83)	
	Unemployed	10.19 (2.92)		10.52 (3.38)		8.66 (2.97)		10.33 (3.63)		39.71 (8.14)	
Education level*	Diploma and low degree	9.77 (2.79)	0.650	11.38 (3.76)	0.487	10.04 (2.61)	0.568	11.30 (3.95)	0.533	42.50 (9.62)	0.990
	Academic Degree	9.69 (2.69)		11.22 (3.50)		10.13 (2.56)		11.45 (3.75)		42.51 (9.13)	
Marital status**	Married	9.64 (2.73)	0.268	11.37 (3.65)	0.047	10.05 (2.63)	0.705	11.33 (3.92)	0.264	42.40 (9.77)	0.194
	Single	9.80 (2.72)		11.06 (3.47)		10.18 (2.47)		11.42 (3.69)		42.48 (8.65)	
	Divorce	10.44 (2.55)		12.76 (3.98)		10.04 (3.02)		12.60 (3.45)		45.84 (7.42)	

*Independents sample t-test; **one-way ANOVA.

Results in **Table 3** showed that people who got information related to mental illness had more social inadequacy (*p* = 0.006), and more self-blame (*p* < 0.001) and these relationships were significant. Also, results revealed that people who did not get information related to mental illness had more help-seeking inhibition (*p* = 0.001). People who reported that they did not know how to get health information had more help-seeking inhibition (*p* = 0.012) (**Table 3**).

**Table 3:** Relationship between demographic variables and self-stigma of depression (SSD).

Variables		SSD *Mean (SD)*									
		Social inadequacy	*P*-value	Help-seeking inhibition	*P*-value	Self-Blame	*P*-value	Shame	*P*-value	SSD	*P*-value
Get information related to mental illness*	Yes	9.86 (2.76)	0.006	11.07 (3.60)	0.001	10.34 (2.47)	< 0.001	11.31 (3.89)	0.228	42.59 (9.39)	0.621
	No	9.33 (2.60)		11.89 (3.53)		9.41 (2.74)		11.63 (3.64)		42.27 (9.11)	
Sources of obtaining health information **	Internet	9.86 (2.64)	0.456	11.37 (3.56)	0.012	10.06 (2.51)	0.052	11.51 (3.83)	0.596	42.82 (9.35)	0.739
	Friends and acquaintances	9.25 (2.77)		11.01 (3.31)		9.60 (2.49)		11.47 (3.36)		41.34 (7.95)	
	Newspapers/magazines	9.82 (2.77)		12.03 (4.28)		9.67 (2.85)		10.03 (4.05)		41.57 (9.69)	
	Radio, television and satellite	9.56 (2.84)		11.48 (3.62)		9.72 (2.68)		11.45 (3.94)		42.22 (9.90)	
	Book	9.42 (2.52)		10.69 (3.38)		10.22 (3.14)		11.13 (3.56)		41.48 (8.29)	
	Physician/Health care providers	9.80 (2.90)		10.90 (3.63)		10.51 (2.52)		11.40 (3.99)		42.62 (9.86)	
	I do not know	9.41 (2.56)		12.89 (3.73)		10 (2.07)		11.13 (3.72)		43.43 (8.03)	
Sources of obtaining information related to mental illness**	Psychologist/Psychiatrist	9.81 (2.94)	0.340	10.33 (3.99)	0.052	10.86 (2.61)	0.191	10.77 (4.42)	< 0.001	41.79 (10.79)	0.008
	Internet	10.02 (2.83)		11.21 (3.50)		10.14 (2.49)		11.51 (3.67)		42.88 (9.19)	
	Physician/Health care providers	10.32 (2.89)		12.12 (4.10)		10.67 (2.81)		12.43 (4.19)		45.56 (11.66)	
	Book	9.51 (2.92)		10.91 (3.15)		10.59 (2.15)		11.13 (3.75)		42.15 (8.14)	
	Friends and acquaintances	9.65 (2.64)		11.54 (3.56)		10 (2.71)		12.28 (3.67)		43.47 (7.94)	
	Radio, television and satellite	9.25 (2.40)		10.44 (3.78)		9.95 (3.06)		9.06 (4.26)		38.72 (10.18)	
	All items	9.84 (2.62)		10.88 (3.49)		10.23 (2.38)		11.06 (3.70)		42.02 (8.68)	
Did you refer to psychologist?*	Yes	10.05 (2.72)	0.068	11.69 (3.77)	0.093	10.32 (2.73)	0.201	10.35 (4.05)	0.878	43.43 (9.88)	0.140
	No	9.65 (2.72)		11.20 (3.56)		10.05 (2.54)		11.40 (3.78)		42.32 (9.19)	
Have you ever had Mental disorder?*	Yes	9.98 (2.79)	0.171	12 (3.85)	0.004	10.26 (2.72)	0.378	11.91 (4.01)	0.052	44.16 (9.91)	0.011
	No	9.67 (2.71)		11.14 (3.53)		10.07 (2.55)		11.30 (3.78)		42.19 (9.17)	
Did your family refer to a psychologist?**	Yes	10.18 (2.64)	0.010	11.65 (4.03)	0.009	10.52 (2.61)	< 0.001	11.78 (4.10)	0.116	44.15 (9.72)	0.031
	No	9.70 (2.72)		11.04 (3.53)		10.18 (2.53)		11.22 (3.83)		42.16 (9.27)	
	I don’t know	9.32 (2.79)		11.85 (3.31)		9.36 (2.62)		11.67 (3.47)		42.21 (8.92)	

* Independents sample T-test, ** One-way ANOVA.

People who reported that they obtained information related to mental illness from physician/healthcare providers had more shame (*p* < 0.001) and SSD (*p* = 0.008). People who reported that they obtained information related to mental illness from radio, television, and satellite had low SSD (*p* = 0.008). There was no observed significant relationship between refer to a psychologist with SSD and its subscales (*P* > 0.05). People who had the history of mental disorder had more help-seeking inhibition (*p* = 0.004) and SSD (*p* = 0.011). People who reported that a member of their family referred to psychologist had more SSD (*p* = 0.031) (**Table 3**). In **Table 4**, participants’ responses to each item of SSD and mean (± SD) of SSD were mentioned. The mean (± SD) of SSD was 42.51 (9.31). In **Table 5**, Pearson correlation between variables were mentioned, and based on the results, there was a positive and significant correlation coefficient between subscales of SSDS (*P* < 0.001). Also, age had only a negative and significant correlation with help-seeking inhibition (*P* < 0.001) (**Table 5**). The results of Independent Samples Effect Sizes of variables were mentioned in **Supplementary Table S1**, and the results of ANOVA Effect Sizes of variables were mentioned in **Supplementary Table S2**.

**Table 4:** Participants’ response to items of self-stigma of depression and mean (± SD) of SSD.

Subscales	Items	*n* (%)					Mean (± SD)
		Strongly disagree	disagree	Not agree and disagree	Agree	Strongly agree	
F1: Social inadequacy	I would feel I couldn’t contribute much socially.	69 (6.4)	213 (19.8)	196 (18.2)	496 (46.1)	101 (9.4)	9.72 (2.72)
	I would feel inadequate around other people.	57 (5.3)	240 (22.3)	254 (23.6)	423 (39.3)	101 (9.4)	
	I would feel like a burden to other people.	70 (6.5)	257 (23.9)	278 (25.9)	380 (35.3)	90 (8.4)	
F2: Help-seeking inhibition	I would think I should be able to cope with things.	157 (14.6)	364 (33.9)	244 (22.7)	250 (23.3)	60 (5.6)	11.28 (3.60)
	I would think I should be able to “pull myself together.”	140 (13)	330 (30.7)	261 (24.3)	279 (26)	65 (6)	
	I would think I should be stronger.	132 (12.3)	360 (33.5)	264 (24.6)	256 (23.8)	63 (5.9)	
	I would think I only had myself to blame.	84 (7.8)	293 (27.3)	329 (30.6)	292 (27.2)	77 (7.2)	
F3: Self-blame	I would think I should be able to cope with things.	51 (4.7)	186 (17.3)	269 (25)	484 (45)	85 (7.9)	10.10 (2.58)
	I would think I should be able to “pull myself together.”	61 (5.7)	192 (17.9)	245 (22.8)	482 (44.8)	95 (8.8)	
	I would think I should be stronger.	59 (5.5)	186 (17.3)	220 (20.5)	454 (42.2)	156 (14.5)	
F4: Shame	I would feel ashamed.	129 (12)	384 (35.7)	254 (23.6)	248 (23.1)	60 (506)	11.39 (3.83)
	I would feel embarrassed.	147 (13.7)	359 (33.4)	254 (23.6)	256 (23.8)	59 (5.5)	
	I would feel inferior to other people	142 (13.2)	342 (31.8)	258 (24)	269 (25)	64 (6)	
	I would feel disappointed in myself.	124 (11.5)	242 (22.5)	209 (19.4)	377 (35.1)	123 (11.4)	
Mean (± SD) of SSD							42.51 (9.31)

**Table 5:** Pearson correlation between variables.

Correlations							
Variables		Age	Social inadequacy	Help-seeking inhibition	Self-Blame	Shame	SSD
Age	Pearson Correlation	1	-.010	.113	-.004	.015	.046
	Sig. (2-tailed)		.751	.000	.889	.619	.133
	*N*	1,075	1,075	1,075	1,075	1,075	1,075
Social inadequacy	Pearson Correlation	-.010	1	.397	.229	.430	.686
	Sig. (2-tailed)	.751		.000	.000	.000	.000
	*N*	1,075	1,075	1,075	1,075	1,075	1,075
Help-seeking inhibition	Pearson Correlation	.113	.397	1	.208	.601	.808
	Sig. (2-tailed)	.000	.000		.000	.000	.000
	*N*	1,075	1,075	1,075	1,075	1,075	1,075
Self-Blame	Pearson Correlation	-.004	.229	.208	1	.231	.520
	Sig. (2-tailed)	.889	.000	.000		.000	.000
	*N*	1,075	1,075	1,075	1,075	1,075	1,075
Shame	Pearson Correlation	.015	.430	.601	.231	1	.833
	Sig. (2-tailed)	.619	.000	.000	.000		.000
	*N*	1,075	1,075	1,075	1,075	1,075	1,075
SSD	Pearson Correlation	.046	.686	.808	.520	.833	1
	Sig. (2-tailed)	.133	.000	.000	.000	.000	
	*N*	1,075	1,075	1,075	1,075	1,075	1,075

## Discussion

The aim of this study was to investigate the state of depression self-stigma and its relationship with demographic factors in the Iranian population in Gonabad city. In general, the results showed that the average self-stigma of depression was 42.51 ± 9.31 from 70. The self-stigma of depression is one of the main obstacles to help seeking and treatment. In general, most studies have shown that the level of self-stigma of depression was moderate (28, 30). In a study by Werner et al., aimed at evaluating self-stigma in the elderly with depression, showed that self-stigma of depression was moderate (30).

Based on the results, there was a significant relationship between age and help-seeking inhibition, and with age, help-seeking inhibition behavior increased, meaning that with age, people are less likely to help seeking. One of the reasons for this may be that by age, people tend to maintain their independence and not interfere with others in their health challenges and consider help-seeking as a threat to their independence (31). Teo et al., in a scoping review among older adults, showed that with age, help-seeking behaviors decrease (31). There was also a connection between age and self-blame, and younger people had more self-blame. In a study by Tanzer, results showed that the self-blame is related to childhood experiences, and because of this, the self-blame is more among young people (32).

The findings revealed a relationship between sex and help-seeking inhibition, self-blame, social inadequacy, shame, and SSD. Males showed more help-seeking inhibition, shame, and SSD than females. One of the reasons for this is the social norms of traditional masculinity in Iran, making these norms difficult for men and making men avoid emotions. On the other hand, help-seeking by males is the possibility of ridiculing or being marginalized by others, which increases their shame and self-stigma (33, 34). Staiger et al., also showed in a qualitative study that men are less likely to help-seeking than women (35). Oliffe’s research indicated that men are more likely to face obstacles when seeking help with mental health difficulties. This reluctance was frequently influenced by social stigma and the perception of weakness associated with asking for assistance (36).

According to the results of this study, people with a history of mental illness had more SSD. In general, studies have shown that self-stigma is common among people with a history of various mental health disorders, including depression, schizophrenia, bipolar disorder, and anxiety disorders (37, 38). Dubreucq et al. conducted a study aimed at examining self-stigma among people with mental health problems and showed that people with a history of mental illness had higher levels of stigma than those without such history (39).

The results of this study showed that social inadequacy had positive and significant correlation with SSD and means that the level of SSD levels increased with increased social inadequacy. Social inadequacy refers to the lack of proper operating in social situations that avoid help-asking by people with depression. So, this sense of social inadequacy can increase self-stigma (40). Various studies have shown that people with depression often experience social inadequacy (28, 40). Manos et al., in a study showed, that people who more internalize their feel of social inadequacy felt more self-stigma (41).

Our findings showed that shame had positive and significant correlation with SSD. Therefore, the amount of SSD increases with increasing shame level. The feeling of shame with the internalization of stereotypes and negative beliefs causes more self-stigma among people with depression. The results of our study were in line with a longitudinal study aimed at investigating the relationship between self-stigma and depression among individuals with schizophrenia-spectrum disorders, and the results showed that shame was the most common aspect of self-stigma (42). Another study was conducted by Oakley et al., showed that women who were less likely to be ashamed of depression reported less self-stigma (43).

The findings of this study indicated that self-blame had positive and significant correlation with SSD, as the SSD level increases as the self-blame increases. The self-blame can lead to a sense of shame, social inadequacy, and help-seeking help-seeking inhibition, and finally led to self-stigma (44). The results of our study were in line with the results of a study that showed patients with depression have been more responded to negative stereotypes and had high self-blame, and, as a result, self-stigma increased in these patients (45).

According to the findings of this study, help-seeking inhibition had positive and significant correlation with SSD, so that with increasing help-seeking inhibition, people experienced more self-stigma. People with more inhibitors are less likely to seek help, which increases self-stigma. The internalization of negative stereotypes and feelings of depression can cause people to be reluctant to seek support or treatment; as a result, self-stigma increases (40). Conceição et al., in a study among college students, showed that help-seeking and self-stigma are related to each other and a decrease in help seeking leads to an increase in self-stigma (46).

This study had strengths and limitations. The study was conducted with a large sample size and used credible and reliable tools. A weakness of this study was that only the relationships between variables were measured. In addition, generalizing the results of this study to other societies and cultures is another limitation of this research.

## Conclusion

The results of the study showed that SSD level was 42.51 ± 9.31 from 70. Since SSD is one of the main obstacles to helping seeking and treatment, it is essential to provide knowledge and awareness in this area. In addition, to effectively reduce stigma around mental health, especially in public health interventions, training for health care providers in this field, social contact interventions, and health communication development are suggested. Furthermore, based on the results, some factors, such as age, sex, previous knowledge, and a history of being referred to a physician or healthcare provider, were associated with SSD. Therefore, considering these variables can help us design more accurate and targeted educational interventions. Although this study identified some factors associated with SSD, more research is needed to fully understand the factors affecting it, including the role of cultural diversity in SSD prevalence.

## Data Availability

The original contributions presented in the study are included in the article/**Supplementary Material**. Further inquiries can be directed to the corresponding author.
