# Corrigendum: The survey of the status of self-stigma of depression and its relationship with demographic factors in Gonabad, Iran

**DOI:** 10.3389/fpsyt.2024.1544260

**Published:** 2025-02-04

**Authors:** Hadi Tehrani, Fatemehzahra Naddafi, Mahbobeh Nejatian, Alireza Jafari

**Affiliations:** ^1^ Social Determinants of Health Research Center, Mashhad University of Medical Sciences, Mashhad, Iran; ^2^ Department of Health Education and Health Promotion, School of Health, Mashhad University of Medical Sciences, Mashhad, Iran; ^3^ Student Research Committee, Gonabad University of Medical Sciences, Gonabad, Iran; ^4^ Social Determinants of Health Research Center, Gonabad University of Medical Sciences, Gonabad, Iran; ^5^ Department of Health Education and Health Promotion, School of Health, Social Development and Health Promotion Research Center, Gonabad University of Medical Sciences, Gonabad, Iran

**Keywords:** self-stigma, depression, stigma, mental health, help seeking

In the published article, there were some errors in **Table 1** as published. Some of the figures listed under the % column were inadvertently miscalculated or written incorrectly in the article. The percents for the first mention of ‘Radio, television and satellite’ was listed as ‘9.8’, ‘Psychologist/Psychiatrist’ was listed as ‘5.5’, second mention of ‘Internet’ was listed as ‘27.6’, the second mention of ‘Physician/Health care providers’ was listed as ‘7.3’, second mention of ‘Book’ was listed as ‘8.3’, second mention of ‘Friends and acquaintances’ was listed as ‘4.3’, the second mention of ‘Radio, television and satellite’ was listed as ‘4’, and ‘All items’ was listed as ‘19.6’. Secondly, the last variable has mistakenly written as ‘Did you family refer to a psychologist?’

The corrected **Table 1** and its caption appears below.

**Table 1:** Characteristics of demographic variables.

Variables		*n* =1,075	
		*n*	%
**Age group**	18-27	432	40.2
	28-37	243	22.6
	38-47	201	18.7
	>47	199	18.5
**Sex**	Male	532	49.5
	Female	543	50.5
**Economic status**	Good	229	21.3
	Medium	711	66.1
	Weak	135	12.6
**Occupation**	Self-employed	162	15.1
	Employed	301	28
	Retired	60	5.6
	Housewife	122	11.3
	labor	29	2.7
	University students	380	35.3
	Unemployed	21	2
**Education level**	Diploma and low degree	401	37.3
	Academic degree	674	62.7
**Marital status**	Married	638	59.3
	Single	412	38.3
	Divorce	25	2.3
**Get information related to mental illness**	Yes	796	74
	No	279	26
**Sources of obtaining health information**	Internet	495	46
	Friends and acquaintances	76	7.1
	Newspapers/ magazines	28	2.6
	Radio, television and satellite	105	9.7
	Book	75	7
	Physician/ Health care providers	250	23.3
	I do not know	46	4.3
**Sources of obtaining information related to mental illness**	Psychologist/Psychiatrist	59	7.2
	Internet	297	36.1
	Physician/ Health care providers	78	9.5
	Book	89	10.8
	Friends and acquaintances	46	5.6
	Radio, television and satellite	43	5.2
	All items	211	25.6
**Did you refer to psychologist?**	Yes	185	17.2
	No	890	82.8
**Have you ever had mental disorder?**	Yes	173	16.1
	No	901	83.9
**Did your family refer to psychologist?**	Yes	185	17.2
	No	710	66
	I don’t know	180	16.7

In the published article, there was an error in **Table 3** as published. The last variable was written as ‘Did you family refer to psychologist?*’. It should read as ‘Did your family refer to a psychologist?**’ The corrected **Table 3** and its caption appears below.

**Table 3:** Relationship between demographic variables and self-stigma of depression (SSD).

Variables		SSD *Mean (SD)*									
		Social inadequacy	P-value	Help-seeking inhibition	P-value	Self-Blame	P-value	Shame	P-value	SSD	P-value
Get information related to mental illness*	Yes	9.86 (2.76)	0.006	11.07 (3.60)	0.001	10.34 (2.47)	<0.001	11.31 (3.89)	0.228	42.59 (9.39)	0.621
	No	9.33 (2.60)		11.89 (3.53)		9.41 (2.74)		11.63 (3.64)		42.27 (9.11)	
Sources of obtaining health information **	Internet	9.86 (2.64)	0.456	11.37 (3.56)	0.012	10.06 (2.51)	0.052	11.51 (3.83)	0.596	42.82 (9.35)	0.739
	Friends and acquaintances	9.25 (2.77)		11.01 (3.31)		9.60 (2.49)		11.47 (3.36)		41.34 (7.95)	
	Newspapers/ magazines	9.82 (2.77)		12.03 (4.28)		9.67 (2.85)		10.03 (4.05)		41.57 (9.69)	
	Radio, television and satellite	9.56 (2.84)		11.48 (3.62)		9.72 (2.68)		11.45 (3.94)		42.22 (9.90)	
	Book	9.42 (2.52)		10.69 (3.38)		10.22 (3.14)		11.13 (3.56)		41.48 (8.29)	
	Physician/ Health care providers	9.80 (2.90)		10.90 (3.63)		10.51 (2.52)		11.40 (3.99)		42.62 (9.86)	
	I do not know	9.41 (2.56)		12.89 (3.73)		10 (2.07)		11.13 (3.72)		43.43 (8.03)	
Sources of obtaining information related to mental illness**	Psychologist/Psychiatrist	9.81 (2.94)	0.340	10.33 (3.99)	0.052	10.86 (2.61)	0.191	10.77 (4.42)	<0.001	41.79 (10.79)	0.008
	Internet	10.02 (2.83)		11.21 (3.50)		10.14 (2.49)		11.51 (3.67)		42.88 (9.19)	
	Physician/ Health care providers	10.32 (2.89)		12.12 (4.10)		10.67 (2.81)		12.43 (4.19)		45.56 (11.66)	
	Book	9.51 (2.92)		10.91 (3.15)		10.59 (2.15)		11.13 (3.75)		42.15 (8.14)	
	Friends and acquaintances	9.65 (2.64)		11.54 (3.56)		10 (2.71)		12.28 (3.67)		43.47 (7.94)	
	Radio, television and satellite	9.25 (2.40)		10.44 (3.78)		9.95 (3.06)		9.06 (4.26)		38.72 (10.18)	
	All items	9.84 (2.62)		10.88 (3.49)		10.23 (2.38)		11.06 (3.70)		42.02 (8.68)	
Did you refer to psychologist?*	Yes	10.05 (2.72)	0.068	11.69 (3.77)	0.093	10.32 (2.73)	0.201	10.35 (4.05)	0.878	43.43 (9.88)	0.140
	No	9.65 (2.72)		11.20 (3.56)		10.05 (2.54)		11.40 (3.78)		42.32 (9.19)	
Have you ever had Mental disorder?*	Yes	9.98 (2.79)	0.171	12 (3.85)	0.004	10.26 (2.72)	0.378	11.91 (4.01)	0.052	44.16 (9.91)	0.011
	No	9.67 (2.71)		11.14 (3.53)		10.07 (2.55)		11.30 (3.78)		42.19 (9.17)	
Did your family refer to a psychologist?**	Yes	10.18 (2.64)	0.010	11.65 (4.03)	0.009	10.52 (2.61)	<0.001	11.78 (4.10)	0.116	44.15 (9.72)	0.031
	No	9.70 (2.72)		11.04 (3.53)		10.18 (2.53)		11.22 (3.83)		42.16 (9.27)	
	I don’t know	9.32 (2.79)		11.85 (3.31)		9.36 (2.62)		11.67 (3.47)		42.21 (8.92)	

* Independents sample T-test, ** One-way ANOVA.

In the published article, there was an error in **Table 5** as published. The following footnotes ‘*Correlation is significant at the 0.01 level (2-tailed)’ and ‘* Independents sample T-test, ** One-way ANOVA’ was mistakenly added. The corrected **Table 5** and its caption appears below.

**Table 5:** Pearson correlation between variables.

Correlations							
Variables		Age	Social inadequacy	Help-seeking inhibition	Self-Blame	Shame	SSD
Age	Pearson Correlation	1	-.010	.113	-.004	.015	.046
	Sig. (2-tailed)		.751	.000	.889	.619	.133
	*N*	1,075	1,075	1,075	1,075	1,075	1,075
Social inadequacy	Pearson Correlation	-.010	1	.397	.229	.430	.686
	Sig. (2-tailed)	.751		.000	.000	.000	.000
	*N*	1,075	1,075	1,075	1,075	1,075	1,075
Help-seeking inhibition	Pearson Correlation	.113	.397	1	.208	.601	.808
	Sig. (2-tailed)	.000	.000		.000	.000	.000
	*N*	1,075	1,075	1,075	1,075	1,075	1,075
Self-Blame	Pearson Correlation	-.004	.229	.208	1	.231	.520
	Sig. (2-tailed)	.889	.000	.000		.000	.000
	*N*	1,075	1,075	1,075	1,075	1,075	1,075
Shame	Pearson Correlation	.015	.430	.601	.231	1	.833
	Sig. (2-tailed)	.619	.000	.000	.000		.000
	*N*	1,075	1,075	1,075	1,075	1,075	1,075
SSD	Pearson Correlation	.046	.686	.808	.520	.833	1
	Sig. (2-tailed)	.133	.000	.000	.000	.000	
	*N*	1,075	1,075	1,075	1,075	1,075	1,075

In the published article, there was an error in **Figure 1** as published. ‘Radio, television and satellite’ was mistakenly calculated as ‘9.8%’. The corrected **Figure 1** and its caption appears below.

**Figure 1:**
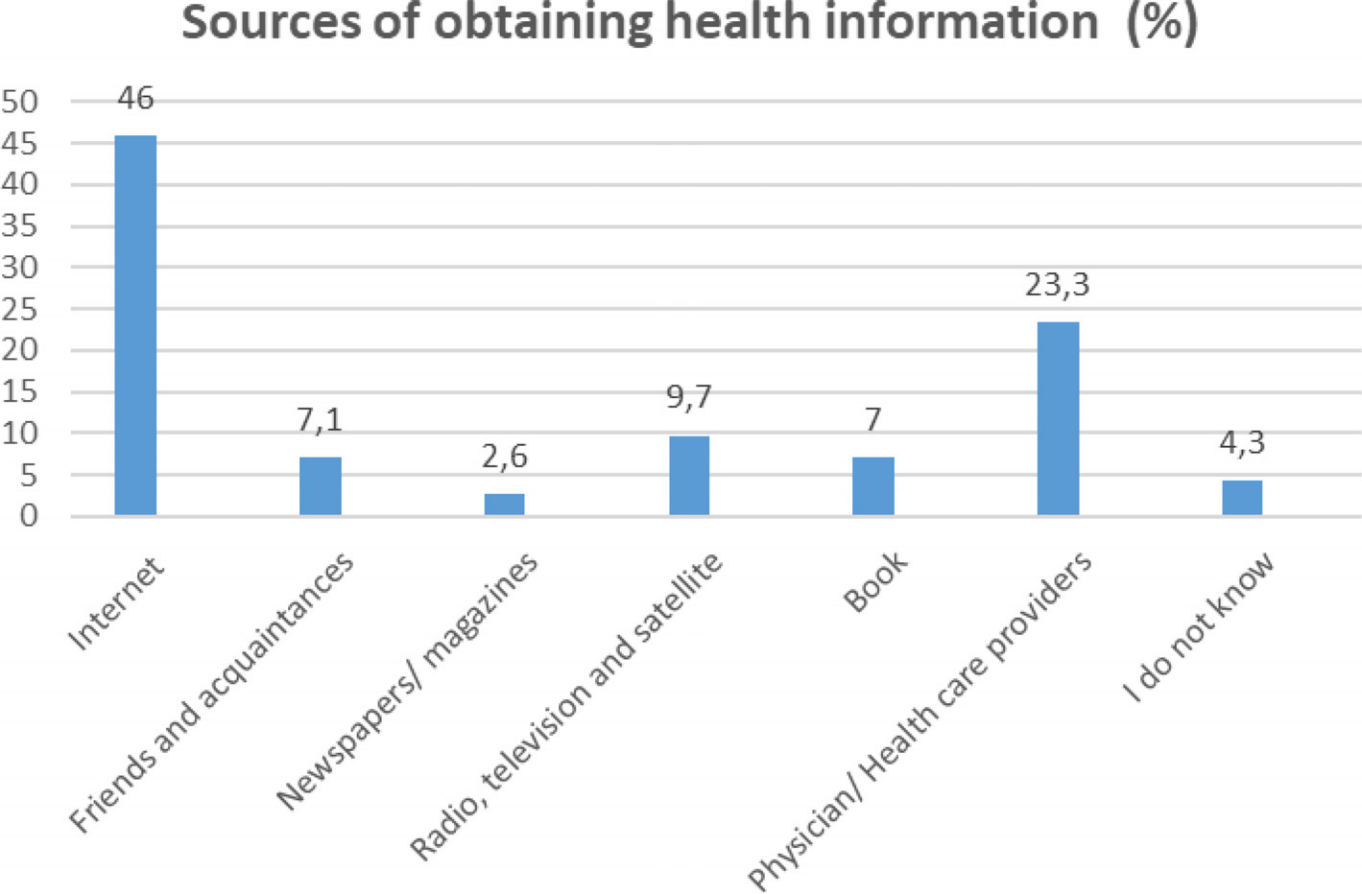
Results of Sources of obtaining health information.

In the published article, there was an error in **Figure 2** as published. The percentages were all miscalculated. For ‘Pyschologist/Psychiatrist’ it was listed as ‘5.5,’ for ‘Internet’ it was listed as ‘27.6’, for ‘Physician/Health care providers’ it was listed as ‘7.3’, for ‘Book’ it was listed as ‘8.3’ and for ‘Friends and acquaintances’ it was listed as ‘4.3’. The corrected **Figure 2** and its caption appears below.

**Figure 2:**
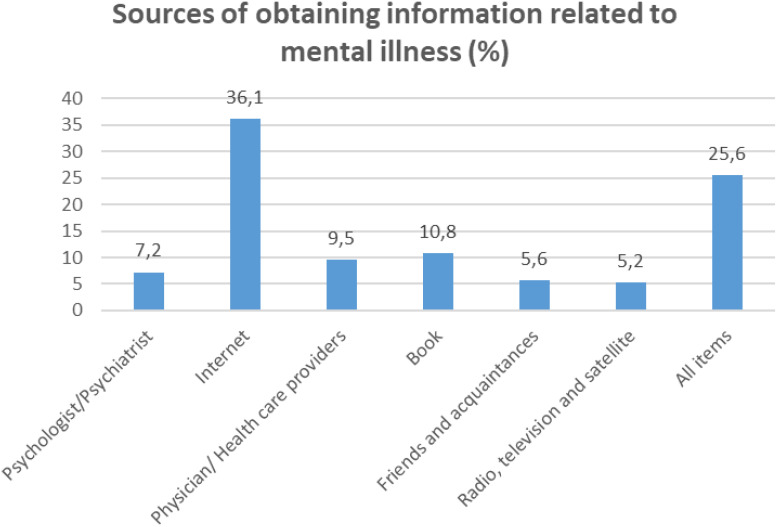
Results of Sources of obtaining information related to mental illness.

The authors apologize for these errors and state that this does not change the scientific conclusions of the article in any way. The original article has been updated.

