# Supplementary material for: The survey of the status of self-stigma of depression and its relationship with demographic factors in Gonabad, Iran
Source: Front Psychiatry. 2024 Nov 28;15:1463879. doi: 10.3389/fpsyt.2024.1463879 (PMC11635171; doi:10.3389/fpsyt.2024.1463879)
Supplement: Supplementary file 1 [file Table1.docx]

**Table S1:** Independent Samples Effect Sizes of variables

| **Variables** | | | **Standardizer** | **Point Estimate** | **95% Confidence Interval** | |
| --- | --- | --- | --- | --- | --- | --- |
|  |  |  |  |  | **Lower** | **Upper** |
| **Sex** | Social inadequacy | Cohen's d | 2.72303 | -.144 | -.264 | -.024 |
|  | Help-seeking inhibition | Cohen's d | 3.54478 | .366 | .246 | .487 |
|  | Self-Blame | Cohen's d | 2.57649 | -.147 | -.267 | -.027 |
|  | Shame | Cohen's d | 3.81624 | .189 | .069 | .309 |
|  | Total of Self-stigma of depression | Cohen's d | 9.30106 | .134 | .015 | .254 |
| **Get information related to mental illness** | Social inadequacy | Cohen's d | 2.72033 | .193 | .057 | .330 |
|  | Help-seeking inhibition | Cohen's d | 3.58602 | -.228 | -.365 | -.091 |
|  | Self-Blame | Cohen's d | 2.55081 | .366 | .229 | .503 |
|  | Shame | Cohen's d | 3.83074 | -.084 | -.220 | .052 |
|  | Total of Self-stigma of depression | Cohen's d | 9.32100 | .034 | -.102 | .171 |
| **Did you refer to psychologist?** | Social inadequacy | Cohen's d | 2.72588 | .148 | -.011 | .306 |
|  | Help-seeking inhibition | Cohen's d | 3.59916 | .136 | -.023 | .294 |
|  | Self-Blame | Cohen's d | 2.58150 | .103 | -.055 | .262 |
|  | Shame | Cohen's d | 3.83330 | -.012 | -.171 | .146 |
|  | Total of Self-stigma of depression | Cohen's d | 9.31262 | .119 | -.039 | .278 |
| **Have you ever had Mental disorder?** | Social inadequacy | Cohen's d | 2.72587 | .114 | -.049 | .276 |
|  | Help-seeking inhibition | Cohen's d | 3.59169 | .239 | .076 | .402 |
|  | Self-Blame | Cohen's d | 2.58309 | .073 | -.090 | .236 |
|  | Shame | Cohen's d | 3.82484 | .161 | -.002 | .324 |
|  | Total of Self-stigma of depression | Cohen's d | 9.29799 | .212 | .049 | .375 |
| **Education level** | Social inadequacy | Cohen's d | 2.72985 | .029 | -.095 | .152 |
|  | Help-seeking inhibition | Cohen's d | 3.60308 | .044 | -.080 | .167 |
|  | Self-Blame | Cohen's d | 2.58308 | -.036 | -.160 | .088 |
|  | Shame | Cohen's d | 3.83264 | -.039 | -.163 | .084 |
|  | Total of Self-stigma of depression | Cohen's d | 9.32206 | -.001 | -.124 | .123 |
