# Supplementary material for: The survey of the status of self-stigma of depression and its relationship with demographic factors in Gonabad, Iran
Source: Front Psychiatry. 2024 Nov 28;15:1463879. doi: 10.3389/fpsyt.2024.1463879 (PMC11635171; doi:10.3389/fpsyt.2024.1463879)
Supplement: Supplementary file 2 [file Table2.docx]

**Table S2:** ANOVA Effect Sizes of variables

| **Variables** | | | **Point Estimate** | **95% Confidence Interval** | |
| --- | --- | --- | --- | --- | --- |
|  |  |  |  | **Lower** | **Upper** |
| **Age group** | Social inadequacy | Eta-squared | .001 | .000 | .004 |
|  | Help-seeking inhibition | Eta-squared | .016 | .003 | .032 |
|  | Self-Blame | Eta-squared | .011 | .001 | .024 |
|  | Shame | Eta-squared | .003 | .000 | .011 |
|  | Total of Self-stigma of depression | Eta-squared | .006 | .000 | .016 |
| **Economic status** | Social inadequacy | Eta-squared | .002 | .000 | .010 |
|  | Help-seeking inhibition | Eta-squared | .006 | .000 | .018 |
|  | Self-Blame | Eta-squared | .001 | .000 | .008 |
|  | Shame | Eta-squared | .003 | .000 | .011 |
|  | Total of Self-stigma of depression | Eta-squared | .002 | .000 | .010 |
| **Occupation** | Social inadequacy | Eta-squared | .004 | .000 | .009 |
|  | Help-seeking inhibition | Eta-squared | .009 | .000 | .018 |
|  | Self-Blame | Eta-squared | .012 | .000 | .022 |
|  | Shame | Eta-squared | .007 | .000 | .014 |
|  | Total of Self-stigma of depression | Eta-squared | .004 | .000 | .009 |
| **Marital status** | Social inadequacy | Eta-squared | .002 | .000 | .010 |
|  | Help-seeking inhibition | Eta-squared | .006 | .000 | .017 |
|  | Self-Blame | Eta-squared | .001 | .000 | .005 |
|  | Shame | Eta-squared | .002 | .000 | .011 |
|  | Total of Self-stigma of depression | Eta-squared | .003 | .000 | .012 |
| **Sources of obtaining health information** | Social inadequacy | Eta-squared | .005 | .000 | .011 |
|  | Help-seeking inhibition | Eta-squared | .015 | .001 | .027 |
|  | Self-Blame | Eta-squared | .012 | .000 | .022 |
|  | Shame | Eta-squared | .004 | .000 | .009 |
|  | Total of Self-stigma of depression | Eta-squared | .003 | .000 | .007 |
| **Sources of obtaining information related to mental illness** | Social inadequacy | Eta-squared | .008 | .000 | .017 |
|  | Help-seeking inhibition | Eta-squared | .015 | .000 | .028 |
|  | Self-Blame | Eta-squared | .011 | .000 | .021 |
|  | Shame | Eta-squared | .033 | .008 | .053 |
|  | Total of Self-stigma of depression | Eta-squared | .021 | .002 | .037 |
| **Did you family refer to psychologist?** | Social inadequacy | Eta-squared | .009 | .001 | .022 |
|  | Help-seeking inhibition | Eta-squared | .009 | .001 | .022 |
|  | Self-Blame | Eta-squared | .019 | .005 | .037 |
|  | Shame | Eta-squared | .004 | .000 | .014 |
|  | Total of Self-stigma of depression | Eta-squared | .006 | .000 | .018 |
